# Incidental findings associated with MRI of the hand and wrist

**DOI:** 10.1093/bjr/tqaf194

**Published:** 2025-08-12

**Authors:** Megan A Beese, Prasant Gurung, Jack A Hall, Alexander Shuttleworth, Shuyi Zhen, Chiraag Karia, Grainne Bourke, Ryckie G Wade

**Affiliations:** Department of Plastic, Reconstructive and Hand Surgery, Leeds Teaching Hospitals Trust, Leeds, LS1 3EX, United Kingdom; Department of Plastic, Reconstructive and Hand Surgery, Leeds Teaching Hospitals Trust, Leeds, LS1 3EX, United Kingdom; Institute of Applied Health Research, University of Birmingham, Birmingham, B15 2TT, United Kingdom; Department of Plastic, Reconstructive and Hand Surgery, Leeds Teaching Hospitals Trust, Leeds, LS1 3EX, United Kingdom; Leeds Institute for Medical Research, University of Leeds, Leeds, LS9 7TF, United Kingdom; Department of Plastic, Reconstructive and Hand Surgery, Leeds Teaching Hospitals Trust, Leeds, LS1 3EX, United Kingdom; Leeds Institute for Medical Research, University of Leeds, Leeds, LS9 7TF, United Kingdom; Department of Plastic, Reconstructive and Hand Surgery, Leeds Teaching Hospitals Trust, Leeds, LS1 3EX, United Kingdom; Leeds Institute for Medical Research, University of Leeds, Leeds, LS9 7TF, United Kingdom; Department of Plastic, Reconstructive and Hand Surgery, Leeds Teaching Hospitals Trust, Leeds, LS1 3EX, United Kingdom; Leeds Institute for Medical Research, University of Leeds, Leeds, LS9 7TF, United Kingdom; The Advanced Imaging Centre, Leeds General Infirmary, Leeds, LS1 3EX, United Kingdom

**Keywords:** hand, wrist, MRI, incidental, incidentaloma

## Abstract

**Objectives:**

To identify the prevalence of incidentalomas and incidental findings in symptomatic patients undergoing MRI of the hand or wrist.

**Methods:**

This retrospective cross-sectional study included all children and adults who completed MRI of the hand or wrist over a 14-year period in a single UK tertiary centre. An incidental finding was any abnormality (structural or signal-based), suspected injury or disease-process that was not already established or suspected. Incidentalomas were defined as incidental findings requiring further investigation or treatment. Marginal standardisation was used to explore relationships between prognostic factors and outcomes.

**Results:**

Overall, 490 out of 2138 (22.9%) scans contained 1 or more incidental anomalies and 67 (3.1%) had at least 1 incidentaloma. The risk of incidentalomas doubled (risk ratio (RR), 1.93; 95% CI, 1.01 to 3.70) when reported by a trainee and reviewed by a consultant compared to a consultant alone; increased by 12% (RR, 1.12; 95% CI, 0.98 to 1.28) per additional decade of life; and were less likely (RR, 0.34; 95% CI, 0.12 to 0.94) when contrast was used. Three incidentalomas were found to be malignant (3.3%).

**Conclusions:**

The risk of incidentalomas and incidental findings in MRI of the hand and wrist is lower than solid organs. Our data may be used to inform patients about the risks of imaging and allow health services to plan the capacity and capability to deal with such events.

**Advances in knowledge:**

One in 4 hand or wrist MRIs yields an incidental finding and out of these, around 1 in 7 required further action.

## Introduction

MRI is a commonly used imaging modality in upper limb clinical work and research^1^ given its unparalleled diagnostic accuracy for a variety of pathologies affecting the hand and wrist.^2^ However, the anatomy of the hand and wrist is complex, containing several small structures, morphologies, and anatomical variations which can present a diagnostic challenge.^1^^,^^3^ As diagnostic imaging technologies advance, the problems surrounding incidental findings and their management has also grown.^4^ Incidental findings may necessitate further medical intervention and, in some cases, early identification is beneficial but equally, irrespective of the ultimate nature of the incidental finding, the process can increase healthcare costs^5^ and patient anxiety.^6^

Despite advancements in medical imaging, informed consent before imaging remains poor.^6^ Consequently, there has been a growing call for more comprehensive research to investigate the potential consequences and outcomes associated with incidental findings.^5^ The prevalence of incidental findings varies considerably by anatomical location and tissue type. Despite an abundance of research on the topic in solid organs, the risks of incidental findings in upper limb imaging remains unknown. The primary objective of this study was to identify the risk of incidental findings which required further investigation or treatment (which we term “incidentalomas”). The secondary objective was to identify the risk of incidental findings in the hand or wrist, irrespective of whether further intervention was required.

## Methods

### Design and setting

This retrospective cross-sectional study identified patients undergoing their first hand or wrist MRI between April 12, 2007 and December 4, 2021, within a single tertiary care centre in the United Kingdom. This sample size was from the beginning of electronic records to the date data collection commenced. This study was conducted as an evaluation of service and as per the United Kingdom Health Research Authority, neither patient consent nor ethical approval was required.

### Participants

We included all symptomatic patients undergoing their first MRI scan of the hand or wrist in the identified study period. Duplicate (sequential) scans were discarded in the search strategy. Later, we excluded imaging from patients who terminated their scan prematurely or when records had no report to form our primary study population. A sensitivity analysis was conducted by including patients who were excluded from the primary analysis due to artefacts or incomplete scans, which is available in Supplementary Materials.

### Data collection

Data was collected verbatim from radiology reports stored on the hospital trust database by four authors (M.A.B., P.G., S.Z., and A.S.) into a predefined and standardised database. To ensure accuracy and consistency of data extraction, a random sample of records were dual extracted by independent authors. We defined an “incidentaloma” as an incidental finding which required further action of any sort, such as additional imaging, blood tests, biopsy, or clinical surveillance appointment. We defined an incidental finding as any clinically relevant abnormality which was not consistent with the indication of the scan. Determinants intended to be explained and potential confounders were extracted from the radiology reports and included age of the patient at the time of scan, biological sex (male, female), the anatomical location of the scan (hand only, wrist only, hand and wrist), the clinical indication of the scan (trauma, further imaging, haematological/vascular pathology, inflammatory conditions, locking/instability, neurological deficit, operative surveillance, tumour), the reporting grade of the radiologists (consultant only, trainee and consultant co-reporting, trainee reporting with review by a consultant, unknown), field strength (1.5T, 3T, missing) and contrast enhanced scan (yes, no). Age was continuous, centred at 40 years and scaled meaning that one point equaled 10 years.

### Statistics

Categorical variables were summarized by count and percentage. Age was summarised as median and interquartile range. The risk of incidentalomas and incidental findings (overall and within strata) were calculated using the exact binomial method.^7^ We explored the effect of prespecified determinants on the risk of incidental findings and incidentalomas using marginal standardisation. In marginal standardisation, multivariable logistic regression is used to predict outcomes for each level of the determinant to be explained and then contrasted to obtain marginal adjusted risk ratios and risk differences, with confidence intervals estimated using the delta method.^8^ Adjustment variables were identified from a directed acyclic graph (DAG) (Figure S1) to estimate the total effect of each determinant. All confidence intervals are at the 95% level. As we did not intend to state statistical significance of the effect of determinants, we did not report *p* values. All analyses were conducted in R version 4.3.2 (R Core Team, 2023). The anonymized raw data and R code are available at https://osf.io/3qpd9/.

## Results

**Figure 1.**
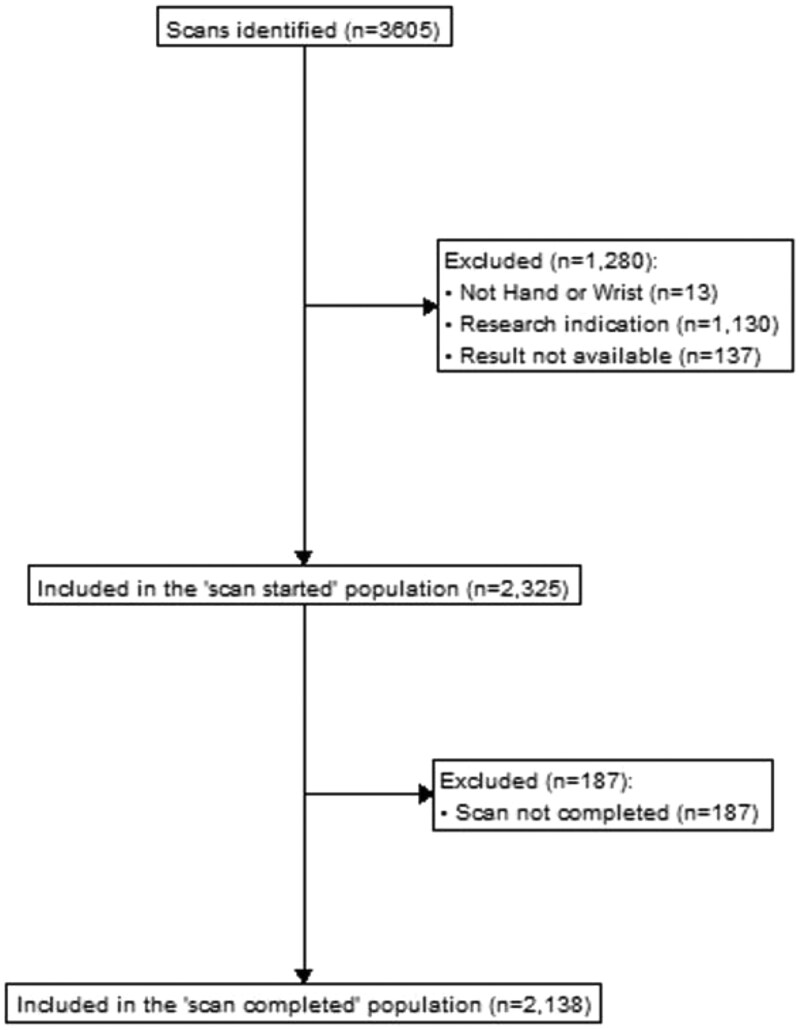
A flowchart of participants in the study.

About 3605 MRI scans of the hand and wrist were identified. A total of 1280 scans were excluded for the following reasons: unreported research scans (*n = *1130), not the hand or wrist (*n = *13), and MRIs imported from other hospitals and lacking a full report (*n = *137). A further 187 incomplete scans and/or containing artefacts preventing complete reporting were excluded. Therefore, a total of 2138 scans from patients were included (Figure 1). Table 1 outlines the baseline characteristics of participants in this study. Overall, 490 scans (22.9%; 95% CI, 21.2% to 24.8%) highlighted at least one incidental finding. A total of 67 out of 2138 (3.1%; 95% CI, 2.4% to 4.0%) were found to have an incidentaloma.

**Table 1.** Baseline characteristics of patients who completed their scan.

	*N = *2138
Age at scan	
Mean (SD)	36.6 (17.3)
Median (Q1, Q3)	34.2 (23.1, 48.7)
Sex	
Female	1107 (51.8%)
Male	1031 (48.2%)
Location	
Hand only	319 (14.9%)
Wrist only	1795 (84.0%)
Hand and wrist	24 (1.1%)
Indication	
Trauma	870 (40.7%)
Further imaging	688 (32.2%)
Haematological/vascular pathology	95 (4.4%)
Inflammatory conditions	197 (9.2%)
Locking/instability	37 (1.7%)
Neurological deficit	21 (1.0%)
Operative planning	70 (3.3%)
Tumour	160 (7.5%)
Reporting grade	
Consultant only	1684 (78.8%)
Co-reporting consultant and trainee	251 (11.7%)
Reported by trainee, reviewed by consultant	203 (9.5%)
Field strength	
1.5	1730 (80.9%)
3	408 (19.1%)
Contrast enhanced scan	
No	1819 (85.1%)
Yes	319 (14.9%)

Table 2 displays the relative rates of incidental findings in the hand and/or wrist. Patients were found to have an increased overall risk of 12% (RR, 1.12; 95% CI, 1.07 to 1.17) of an incidental finding on MRI per additional decade of age. The risk of identifying an incidental finding on wrist MRI increased by 71% (adjusted risk ratio (aRR), 1.71; 95% CI, 1.30 to 2.24) in comparison to scanning the hand only. The use of intravenous contrast decreased the risk of incidental findings by 16% (aRR, 0.84; 95% CI, 0.65 to 1.07). The risk of incidental findings increased by 50% (aRR, 1.50; 95% CI, 1.22 to 1.85) when trainees co-reported with consultants and by 61% (aRR, 1.61; 95% CI, 1.30 to 2.00) when reported by a trainee and later reviewed by a consultant.

**Table 2.** Relative rates of incidental findings.

	Scans with at least one incidental finding/total scans; % (95% CI)	Unadjusted estimate (95% CI)	Adjusted estimate (95% CI)
Overall	490/2138 22.9% (21.2% to 24.8%)		
Age			
Per additional 10 years		RR: 1.12 (1.07 to 1.17) RD: 0.025 (0.015 to 0.035)	
Sex			
Female	254/1107 22.9% (20.5% to 25.5%)	Reference	
Male	236/1031 22.9% (20.4% to 25.6%)	RR: 1.00 (0.85 to 1.17) RD: -0.001 (−0.036 to 0.035)	
Anatomical location			
Hand only	48/319 15.0% (11.3% to 19.5%)	Reference	Reference
Wrist only	440/1795 24.5% (22.5% to 26.6%)	RR: 1.63 (1.24 to 2.14) RD: 0.095 (0.051 to 0.139)	aRR^a^: 1.71 (1.30 to 2.24) aRD^a^: 0.102 (0.059 to 0.145)
Hand and wrist	2/24 8.3% (1.03% to 27.0%)	RR: 0.554 (0.143 to 2.14) RD: −0.067 (−0.184 to 0.050)	aRR^a^: 0.57 (0.15 to 2.21) aRD^a^: −0.062 (−0.178 to 0.054)
Indication			
Trauma	219/870 25.2% (22.3% to 28.2%)	Reference	Reference
Further imaging	138/668 20.1% (17.1% to 23.2%)	RR: 0.80 (0.66 to 0.96) RD: −0.051 (−0.093 to −0.010)	aRR^a^: 0.81 (0.67 to 0.97) aRD^a^: −0.050 (−0.091 to −0.008)
Haematological/vascular pathology	15/95 15.8% (9.1% to 24.7%)	RR: 0.63 (0.39 to 1.01) RD: −0.094 (−0.173 to −0.015)	aRR^a^: 0.63 (0.39 to 1.01) aRD^a^: −0.094 (−0.173 to −0.015)
Inflammatory conditions	45/197 28.4% (22.2% to 35.3%)	RR: 1.129 (0.88 to 1.45) RD: 0.033 (−0.037 to 0.102)	aRR^a^: 1.07 (0.83 to 1.37) aRD^a^: 0.017 (−0.051 to 0.085)
Locking/instability	7/37 18.9% (8.0% to 35.2%)	RR: 0.75 (0.38 to 1.48) RD: −0.063 (−0.192 to 0.067)	aRR^a^: 0.76 (0.39 to 1.48) aRD^a^: −0.062 (−0.192 to 0.069)
Neurological deficit	7/21 33.3% (14.6% to 57.0%)	RR: 1.32 (0.72 to 2.45) RD: 0.082 (−0.122 to 0.285)	aRR^a^: 1.29 (0.70 to 2.39) aRD^a^: 0.075 (−0.126 to 0.276)
Operative planning	15/70 21.4% (12.5% to 32.9%)	RR: 0.85 (0.54 to 1.35) RD: −0.037 (−0.138 to 0.063)	aRR^a^: 0.86 (0.54 to 1.35) aRD^a^: −0.036 (−0.137 to 0.064)
Tumour	33/160 20.6% (14.6% to 27.7%)	RR: 0.82 (0.59 to 1.13) RD: −0.046 (−0.115 to 0.024)	aRR^a^: 0.75 (0.54 to 1.04) aRD^a^: −0.064 (−0.130 to 0.001)
Reporting grade			
Consultant only	344/1684 20.4% (18.5% to 22.4%)	Reference	Reference
Co-reporting consultant and trainee	77/251 30.7% (25.0% to 36.8%)	RR: 1.50 (1.22 to 1.85) RD: 0.102 (0.042 to 0.163)	aRR^b^: 1.50 (1.22 to 1.85) aRD^b^: 0.103 (0.043 to 0.164)
Reported by trainee, reviewed by consultant	69/203 34.0% (27.5% to 41.0%)	RR: 1.66 (1.34 to 2.06) RD: 0.136 (0.068 to 0.204)	aRR^b^: 1.61 (1.30 to 2.00) aRD^b^: 0.126 (0.059 to 0.193)
Field strength			
1.5T	368/1730 21.3% (19.4% to 23.3%)	Reference	Reference
3T	122/408 29.9% (25.5% to 34.6%)	RR: 1.41 (1.18 to 1.67) RD: 0.086 (0.038 to 0.135)	aRR^c^: 1.29 (1.08 to 1.54) aRD^c^: 0.063 (0.016 to 0.109)
Contrast enhanced scan			
No	431/1819 23.7% (21.8% to 25.7%)	Reference	Reference
Yes	59/319 18.5% (14.4% to 23.2%)	RR: 0.78 (0.61 to 0.997) RD: −0.052 (−0.099 to −0.005)	aRR^d^: 0.84 (0.65 to 1.07) aRD^d^: −0.038 (−0.088 to 0.012)

a Adjusted for age and sex.

b Adjusted for age, indication, anatomical location, and field strength.

c Adjusted for age, indication, anatomical location, and reporting grade.

d Adjusted for indication and anatomical location.

Abbreviations: aRD = adjusted risk difference, aRR = adjusted risk ratio, RD = risk difference, RR = risk ratio.

**Figure 2.**
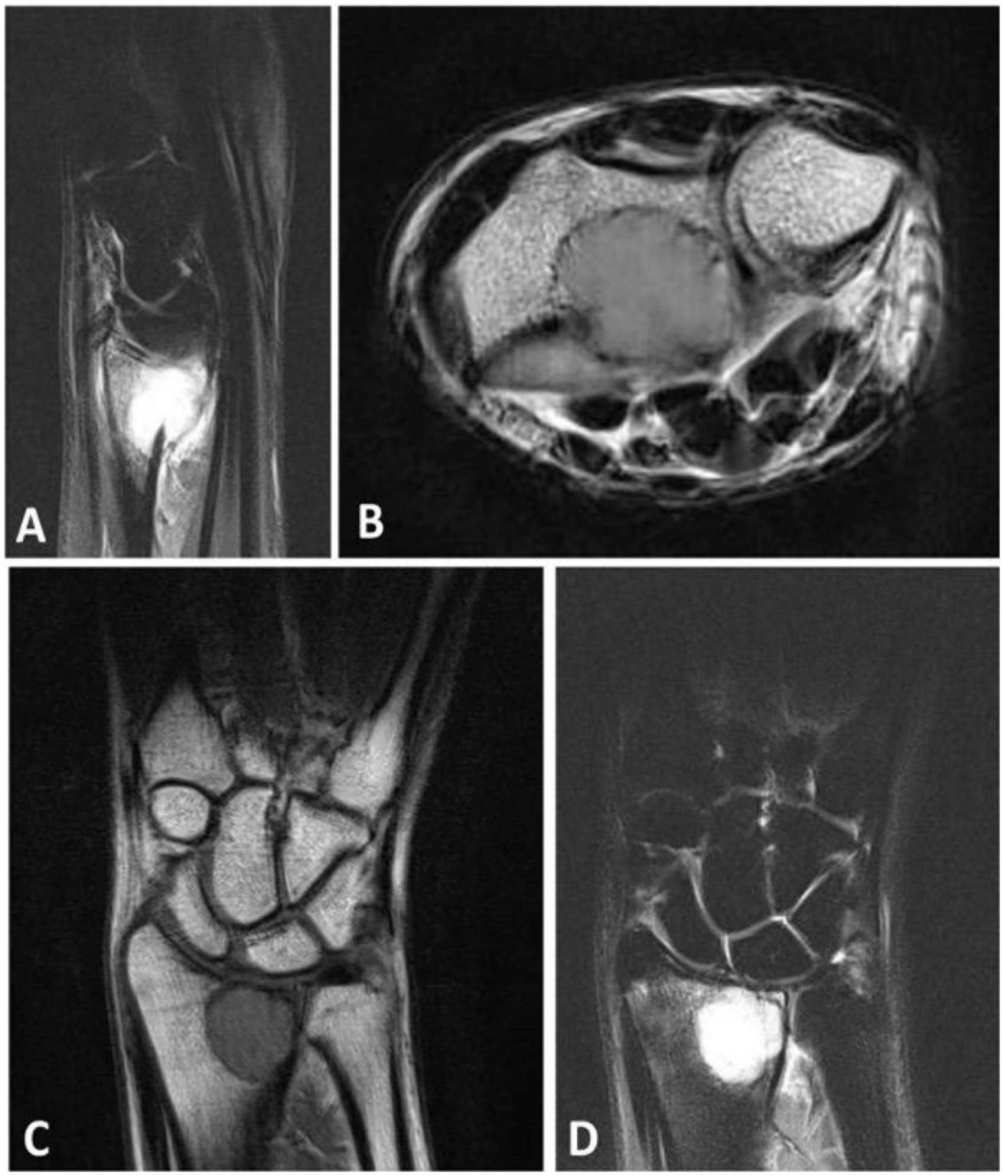
Following a fall from standing height during her first trimester of pregnancy, this 24-year-old female complained of wrist pain for several weeks. A fracture through a lucency was detected on standard X-ray radiography. However, MRI showed a well-defined lesion in the metaphysis of the radius based on T2w (A and D) and T1w (B and C) imaging. This was curettaged during her second trimester with a histological diagnosis of a giant cell tumour.

**Figure 3.**
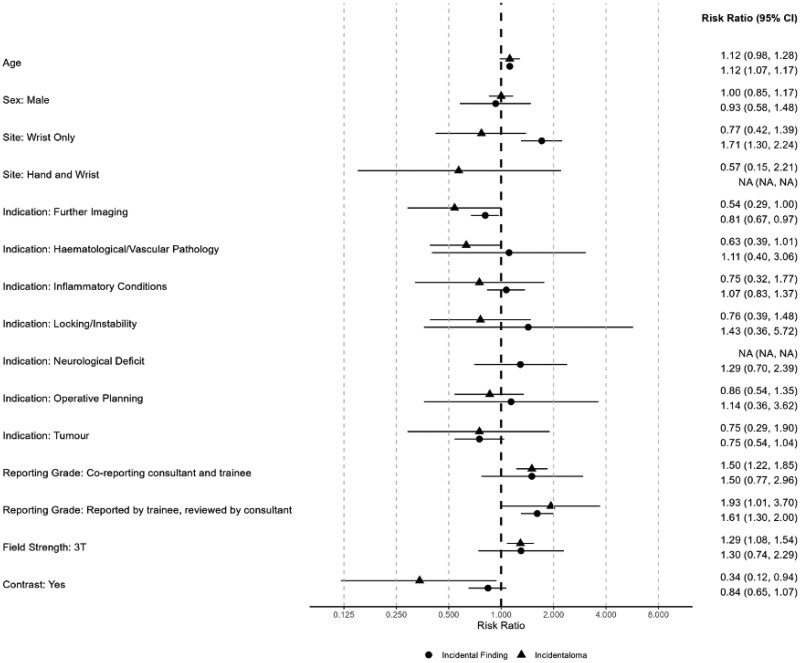
Forest plot of adjusted risk ratios for incidental findings and incidentalomas.

Figure 2 shows an example of an incidentaloma. The risk of incidentalomas increased by 93% (aRR, 1.93; 95% CI, 1.01 to 3.70) when scans were reported by a trainee and reviewed by a consultant compared to when reported by a consultant alone (Table 3). Additionally, the risk of identifying an incidentaloma decreased by 66% (aRR, 0.34; 95% CI, 0.12 to 0.94) when intravenous contrast was used during the MRI scan. MRI scans of the hand had shown an increased risk of incidentalomas findings compared to scanning the wrist alone (aRR, 0.77; 95% CI, 0.42 to 1.39). Trends showed an overall increased risk of 12% (RR, 1.12; 95% CI, 0.98 to 1.28) per decade of age. The adjusted risk ratios and confidence intervals can be seen as a forest plot in Figure 3.

**Table 3.** Relative rates of incidentalomas.

	Incidentalomas/total scans % (95% CI)	Unadjusted estimate (95% CI)	Adjusted estimate (95% CI)
Overall	67/2138 3.1% (2.4% to 4.0%)		
Age			
Per additional 10 years		RR: 1.12 (0.98 to 1.28) RD: 0.004 (−0.001 to 0.008)	
Sex			
Female	36/1107 3.3% (2.3% to 4.5%)	Reference	
Male	31/1031 3.0% (2.1% to 4.2%)	RR: 0.93 (0.58 to 1.48) RD: −0.002 (−0.017 to 0.012)	
Anatomical location			
Hand only	13/319 4.1% (2.2% to 6.9%)	Reference	Reference
Wrist only	54/1795 3.0% (2.3% to 3.9%)	RR: 0.74 (0.41 to 1.34) RD: −0.011 (−0.034 to 0.012)	aRR^a^: 0.77 (0.42 to 1.39) aRD^a^: −0.009 (−0.032 to 0.013)
Hand and wrist	0/24 0.0% (0.0% to 14.2%)	RR: Not computable RD: −0.041 (−0.062 to −0.019)	aRR^a^: Not computable aRD^a^: −0.039 (−0.061 to −0.018)
Indication			
Trauma	33/870 3.8% (2.6% to 5.3%)	Reference	Reference
Further imaging	14/688 2.0% (1.1% to 3.4%)	RR: 0.54 (0.29 to 0.99) RD: −0.018 (−0.034 to −0.001)	aRR^a^: 0.54 (0.29 to 1.00) aRD^a^: −0.018 (−0.034 to −0.001)
Haematological/vascular pathology	4/95 4.2% (1.2% to 10.4%)	RR: 1.11 (0.40 to 3.07) RD: 0.004 (−0.038 to 0.047)	aRR^a^: 1.11 (0.40 to 3.06) aRD^a^: 0.004 (−0.039 to 0.047)
Inflammatory conditions	6/197 3.0% (1.1% to 6.5%)	RR: 0.80 (0.34 to 1.89) RD: −0.007 (−0.035 to 0.020)	aRR^a^: 0.75 (0.32 to 1.77) aRD^a^: -0.010 (−0.036 to 0.017)
Locking/instability	2/37 5.4% (0.7% to 18.2%)	RR: 1.43 (0.36 to 5.72) RD: 0.016 (−0.058 to 0.090)	aRR^a^: 1.43 (0.36 to 5.72) aRD^a^: 0.017 (−0.058 to 0.092)
Neurological deficit	0/21 0.0% (0.0% to 16.1%)	RR: Not computable RD: −0.038 (−0.051 to −0.025)	aRR^a^: Not computable aRD^a^: −0.038 (-0.051 to −0.026)
Operative planning	3/70 4.3% (0.9% to 12.0%)	RR: 1.13 (0.36 to 3.59) RD: 0.005 (−0.044 to 0.054)	aRR^a^: 1.14 (0.36 to 3.62) aRD^a^: 0.005 (−0.045 to 0.055)
Tumour	5/160 3.1% (1.0% to 7.1%)	RR: 0.82 (0.33 to 2.08) RD: −0.007 (−0.037 to 0.023)	aRR^a^: 0.75 (0.29 to 1.90) aRD^a^: −0.010 (−0.039 to 0.018)
Reporting grade			
Consultant only	46/1684 2.7% (2.0% to 3.6%)	Reference	Reference
Co-reporting consultant and trainee	10/251 4.0% (1.9% to 7.2%)	RR: 1.46 (0.75 to 2.85) RD: 0.013 (−0.013 to 0.038)	aRR^b^: 1.50 (0.765 to 2.96) aRD^b^: 0.014 (−0.013 to 0.040)
Reported by trainee, reviewed by consultant	11/203 5.4% (2.7% to 9.5%)	RR: 1.98 (1.04 to 3.77) RD: 0.027 (−0.005 to 0.059)	aRR^b^: 1.93 (1.01 to 3.70) aRD^b^: 0.026 (−0.006 to 0.057)
Field strength			
1.5T	51/1730 2.9% (2.2% to 3.9%)	Reference	Reference
3T	16/408 3.9% (2.3% to 6.3%)	RR: 1.33 (0.77 to 2.31) RD: 0.010 (−0.011 to 0.030)	aRR^c^: 1.30 (0.74 to 2.29) aRD^c^: 0.009 (−0.012 to 0.030)
Contrast enhanced scan			
No	63/1819 3.5% (2.7%-4.4%)	Reference	Reference
Yes	4/319 1.3% (0.3%-3.2%)	RR: 0.36 (0.13 to 0.99) RD: −0.022 (−0.037 to −0.007)	aRR^d^: 0.34 (0.12 to 0.94) aRD^d^: −0.023 (−0.038 to −0.009)

a Adjusted for age and sex.

b Adjusted for age, indication, anatomical location, and field strength.

c Adjusted for age, indication, anatomical location, and reporting grade.

d Adjusted for indication and anatomical location.

Abbreviations: aRD = adjusted risk difference, aRR = adjusted risk ratio, RD = risk difference, RR = risk ratio.

**Figure 4.**
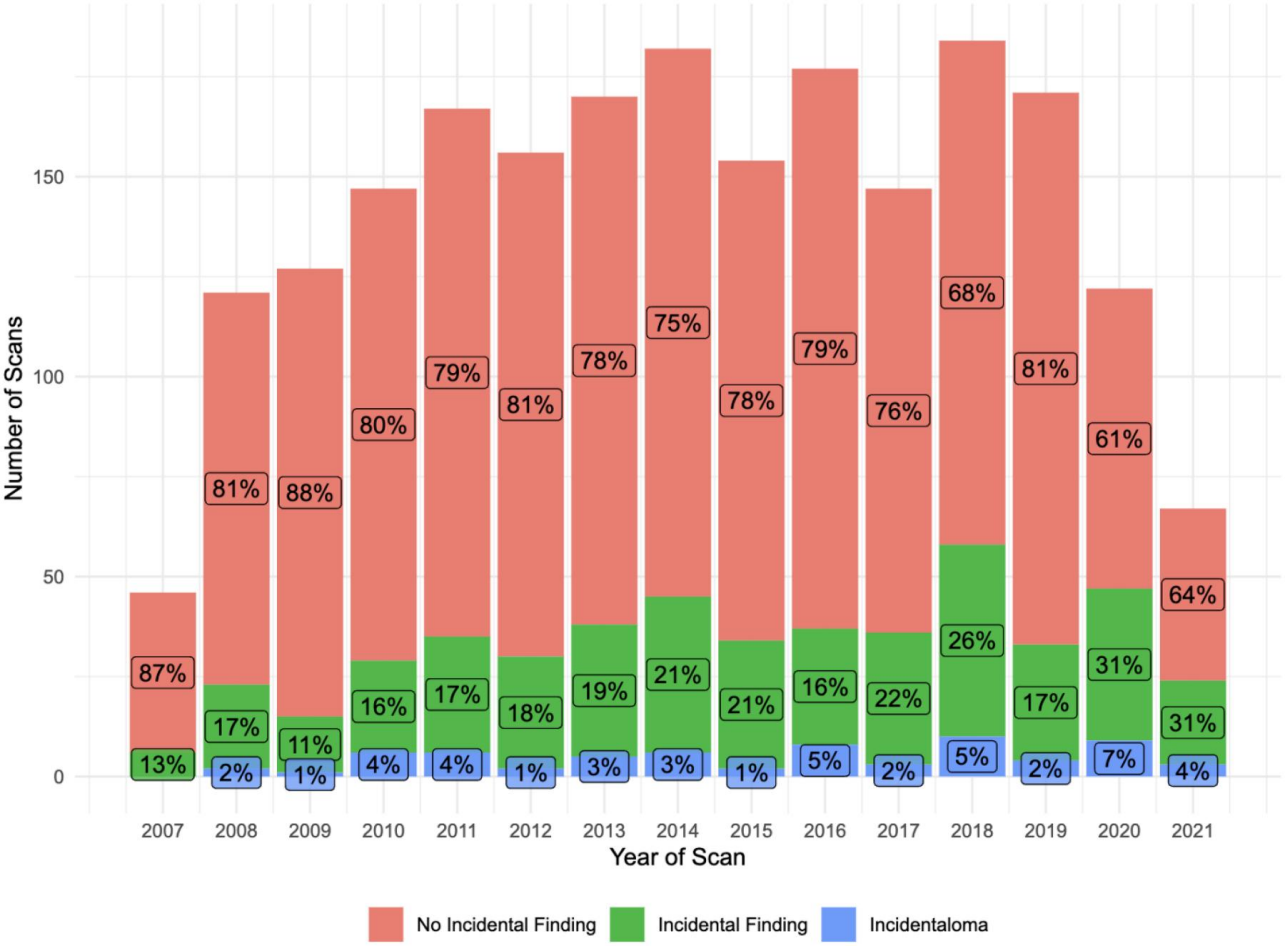
A bar chart showing the proportion of incidental findings and incidentalomas identified over time.

The rate of incidentalomas fluctuated over time (Figure 4). The highest rate of incidentalomas was found to be in 2020 (7.4%) and lowest in 2007 (0%), although data collection started in April 2007. There were 91 incidentalomas identified in 67 scans. Incidentalomas were most commonly found in bone (*n = *35), followed by the joint (*n = *33), muscle (*n = *9), mixed tissue (*n = *9), nerve (*n = *3), and vessel (*n = *2). Sixty-six patients out of 67 that had at least 1 incidentaloma identified required ongoing clinical surveillance, 13 (19%) required further procedures, 7 (10%), 20 (30%) and 5 (7.5%) required a further MRI scan, ultrasound scan, or CT scan, respectively. Additionally, 9 (13%) required blood tests. Three (3%) of incidentalomas were found to be malignant, 5 (6%) benign neoplasms, and 83 (91%) were innocent anomalies which included findings such as ganglion cysts and anatomical variations.

Patients with incidentalomas identified within the joint demonstrated the highest imaging follow-up burden (*n = *22) of which 9 (41%) required follow-up ultrasound, 9 (41%) required another MRI, and 1 (5%) underwent CT. Incidentalomas identified within the bone showed an imaging follow-up rate of 6 (21%) requiring ultrasound, 6 (21%) requiring MRI, and 4 (14%) required CT scans.

## Discussion

The prevalence and burden of incidentalomas and incidental findings on MRI of the hand or wrist are poorly understood in comparison to other anatomical sites. This study aims to identify the prevalence of such findings in symptomatic patients who underwent MRI of the hand or wrist. This study demonstrates that approximately 22.9% of these scans yielded at least 1 incidental finding, of which 13.6% were incidentalomas. Overall, the risk of an incidentaloma was 3.1%.

Our results demonstrate several factors associated with an increased incidence of both incidental findings and incidentalomas. The use of higher field strength scanners (3T *vs*. 1.5T) increased the relative rates of detection of incidental findings and incidentalomas by 29% and 30% (absolutely by 6.3% and 0.9% points), respectively. With higher field strength scanners comes greater sensitivity and signal-to-noise ratio, leading to clearer images. This is in keeping with previous documented literature on brain MRIs which reported that the likelihood of detecting incidental findings was greater in higher resolution sequences.^9^

Orme et al^10^ showed that the frequency of incidental findings varied significantly by body region. The prevalence of incidentalomas in the hand and wrist were 3.1%, which is lower than other sites where major organs are captured within the field-of-view, such as 18% brachial plexus,^1^ 29% breast,^11^ 24% heart,^5^ 12% liver,^12^ and 22% brain and spine.^5^ Wrist MRI scans were found to have almost twice as many incidental findings compared to MRIs of the hand, with 1 additional incidental finding per 10 scans. The wrist is perhaps one of the most complex joints in the body,^2^ it could therefore be hypothesized that the wrist is left prone to a greater range of disorders and pathology. Furthermore, MRI scans of the entire hand are less common given that larger fields-of-view typically demands a lower resolution, hindering the detection of abnormalities.^2^ In line with previous studies,^10^ the risk of incidental findings in the hand and/or wrist increased by 2.5 percentage points per each additional decade of life. These findings could be related to the cumulative risk of various diseases associated with advancing age.

In line with previous literature, this study demonstrates that the grade of the reporting radiologist also affected prevalence.^1^ Scans reported by both a trainee and a consultant radiologist contained incidental findings and incidentalomas more often than scans reported by consultants alone. It could be hypothesised that trainees are more likely to report findings that experienced consultants would have considered clinically irrelevant. Additionally, co-reporting with consultants could lead to more time and discussions surrounding scans.

We are unable to quantify and explain what would have influenced different clinicians’ decision to act on incidental findings. However, our methods represent real-world practice and this carries translational value. Data were collected by 4 independent authors, based around the interpretation of multiple datasets including scans, reports, and clinic letters. Despite a standardised extraction tool, discrepancies may have occurred. To reduce the risk of this bias, author M.A.B. dual extracted a random selection of cases (25%) to evaluate consistency. Incidental findings were less common when the indication for the scan was “further imaging,” in comparison to the reference group of patients with trauma. Since the term “further imaging” carries ambiguity, some findings may have been reported under this umbrella rather than being classified as an incidental finding. To avoid any bias in data, the clinical indication for requesting the MRI as well as pre and postscan clinic letters were reviewed. A directed acyclic graph (DAG) was created (available in Supplementary Materials) to identify appropriate adjustment covariates. This study was conducted using data from a large UK teaching hospital which is both a major trauma and tertiary care centre, so the findings may not be universally generalizable. Furthermore, we did not formally investigate the potential time-confounding in any models, but it could be hypothesized that factors such as an ageing population and the amount of trainees reporting could have had an impact on the rise in incidental findings. It is plausible that the prevalence of incidental findings is lower in healthy volunteers, so it is unclear whether our data are transferable to this population but can be applied in the context of consenting symptomatic patients.

Our results have shown that just under 1 in 4 hand and wrist MRI scans yield an incidental finding, of which 14% (3% overall) required additional investigations or treatments. Incidental findings pose both a financial and emotional burden to patients as well as the healthcare systems involved.^6^ Knowledge of the prevalence and clinical relevance of incidental findings is necessary to aid the associated discussions with patients.^13^ The current guidance on the management of incidental findings is limited and inconsistent.^14^ All doctors and other members of the healthcare team have a “duty of care” to inform patients about clinically significant findings; however, the legal precedent is unclear for research patients or when the findings are of unknown relevance.^15^ More robust clinical frameworks on the management of incidental findings, coupled with increased education for clinicians to improve the consenting process would prove beneficial for patient care. We therefore echo calls from others in the field who advocate for improved and clearer standards on the consent process and the management of incidental findings.^14^

## Supplementary Material

tqaf194_Supplementary_Data

## Data Availability

Data generated by the authors or analysed during the study are available at: https://osf.io/3qpd9/. This is available indefinitely and is publicly available. All patient identifiable data has been removed.

## References

[1] Perumal AR , Anyamele UA, Bhogal RK, et al Incidental findings associated with magnetic resonance imaging of the brachial plexus. Br J Radiol. 2021;94:20200921. 33156721 10.1259/bjr.20200921 PMC7774680

[2] Vassa R , Garg A, Omar IM. Magnetic resonance imaging of the wrist and hand. Pol J Radiol. 2020;85:e461-e488. 32999697 10.5114/pjr.2020.99034 PMC7509702

[3] Wade RG , Takwoingi Y, Wormald JCR, et al MRI for detecting root avulsions in traumatic adult brachial plexus injuries: a systematic review and meta-analysis of diagnostic accuracy. Radiology. 2019;293:125-133. 31429680 10.1148/radiol.2019190218

[4] Stone JH. Incidentalomas—clinical correlation and translational science required. N Engl J Med. 2006;354:2748-2749. 16807411 10.1056/NEJMp058264

[5] O'Sullivan JW , Muntinga T, Grigg S, Ioannidis JPA. Prevalence and outcomes of incidental imaging findings: umbrella review. BMJ. 2018;361:k2387. 29914908 10.1136/bmj.k2387 PMC6283350

[6] Kole J , Fiester A. Incidental findings and the need for a revised informed consent process. AJR Am J Roentgenol. 2013;201:1064-1068. 23902536 10.2214/AJR.13.11138

[7] Collett D. Statistical inference for binary data. In: Modelling Binary Data. 1st ed. Chapman and Hall; 1991:24.

[8] Muller CJ , MacLehose RF. Estimating predicted probabilities from logistic regression: different methods correspond to different target populations. Int J Epidemiol. 2014;43:962-970. 24603316 10.1093/ije/dyu029 PMC4052139

[9] Morris Zoe , Whiteley William N, Longstreth W T, et al Incidental findings on brain magnetic resonance imaging: systematic review and meta-analysis. BMJ. 2009;339:b3016. 19687093 10.1136/bmj.b3016 PMC2728201

[10] Orme NM , Fletcher JG, Siddiki HA, et al Incidental findings in imaging research: evaluating incidence, benefit, and burden. Arch Intern Med. 2010;170:1525-1532. 20876402 10.1001/archinternmed.2010.317 PMC3721142

[11] Liberman L , Morris EA, Dershaw DD, Abramson AF, Tan LK. Ductal enhancement on MR imaging of the breast. AJR Am J Roentgenol. 2003;181:519-525. 12876038 10.2214/ajr.181.2.1810519

[12] Knox M , Slanetz P, Phillips J, et al Incidental liver lesions seen on breast MRI: when is additional imaging warranted? Eur J Radiol. 2017;95:319-324. 28987687 10.1016/j.ejrad.2017.08.018

[13] Langner S , Buelow R, Fleck S, Angermaier A, Kirsch M. Management of intracranial incidental findings on brain MRI. Rofo. 2016;188:1123-1133. 27433969 10.1055/s-0042-111075

[14] Booth TC , Jackson A, Wardlaw JM, Taylor SA, Waldman AD. Incidental findings found in ‘healthy’ volunteers during imaging performed for research: current legal and ethical implications. Br J Radiol. 2010;83:456-465. 20335427 10.1259/bjr/15877332 PMC3473586

[15] Wardlaw J M , Davies H, Booth T C, et al Acting on incidental findings in research imaging. BMJ. 2015;351:h5190. 26556813 10.1136/bmj.h5190

