# Supplementary material for: Incidental findings associated with MRI of the hand and wrist
Source: Br J Radiol. 2025 Aug 12;98(1175):1997–2004. doi: 10.1093/bjr/tqaf194 (PMC12659746; doi:10.1093/bjr/tqaf194)
Supplement: tqaf194_Supplementary_Data [file tqaf194_supplementary_data.zip › Supplementary Figure 2 only.pdf]

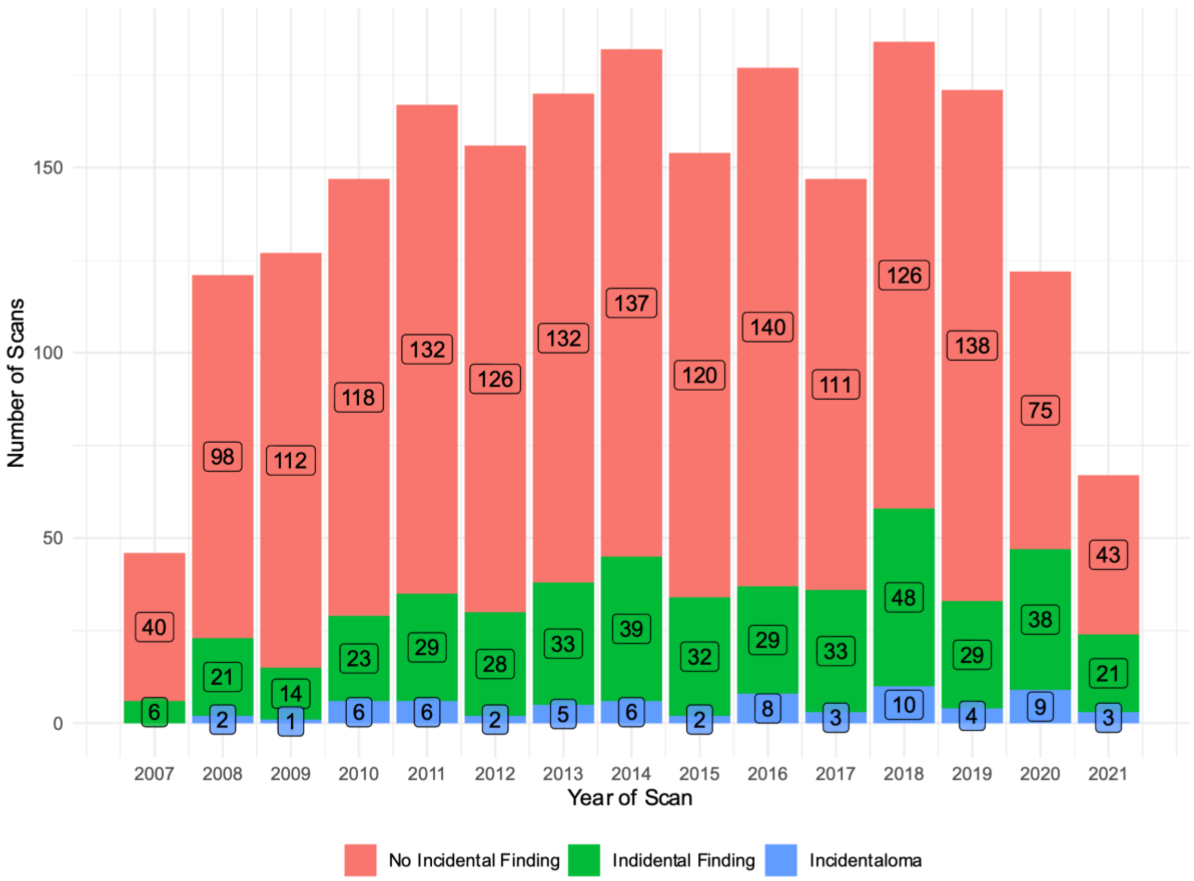

Supplementary Figure 2: A bar chart showing number of scans with proportions of incidental findings and incidentalomas over time.
