# Supplementary material for: Incidental findings associated with MRI of the hand and wrist
Source: Br J Radiol. 2025 Aug 12;98(1175):1997–2004. doi: 10.1093/bjr/tqaf194 (PMC12659746; doi:10.1093/bjr/tqaf194)
Supplement: tqaf194_Supplementary_Data [file tqaf194_supplementary_data.zip › Supplementary table 1 only.docx]

|  |  | Scans with at least one incidental finding/Total Scans  % (95% CI) | Unadjusted Estimate (95% CI) | Adjusted Estimate (95% CI) |  |
| --- | --- | --- | --- | --- | --- |
| Overall | | 527/2325  22.7% (21.0%, 24.4%) |  |  |  |
| Age | |  |  |  |  |
|  | Per additional 10 years |  | RR: 1.11 (1.06, 1.15)  RD: 0.023 (0.013, 0.032) |  |  |
| Sex | |  |  |  |  |
|  | Female | 275/1208  22.8% (20.4%, 25.2%) | Reference |  |  |
|  | Male | 252/1117  22.6% (20.1%, 25.1%) | RR: 0.99 (0.85, 1.15)  RD: -0.002 (-0.036, 0.032) |  |  |
| Anatomical Location | |  |  |  |  |
|  | Hand only | 49/340  14.4% (10.9%, 18.6%) | Reference | Reference |  |
|  | Wrist only | 476/1960  24.3% (22.4%, 26.2%) | RR: 1.69 (1.29, 2.21)  RD: 0.099 (0.058, 0.141) | aRR^1^: 1.76 (1.34, 2.31)  aRD^1^: 0.106 (0.065, 0.146) |  |
|  | Hand and Wrist | 2/25  8.0% (1.0%, 26.0%) | RR: 0.56 (0.14, 2.15)  RD: -0.064 (-0.177, 0.049) | aRR^1^: 0.59 (0.15, 2.26)  aRD^1^: -0.057 (-0.171, 0.056) |  |
| Indication | |  |  |  |  |
|  | Trauma | 244/976  25.0% (22.3%, 27.8%) | Reference | Reference |  |
|  | Further Imaging | 147/722  20.4% (17.5%, 23.5%) | RR: 0.81 (0.68, 0.98)  RD: -0.046 (-0.086, -0.006) | aRR^1^: 0.83 (0.69, 0.99)  aRD^1^: -0.044 (-0.084, -0.003) |  |
|  | Haematological/Vascular Pathology | 15/97  15.5% (8.9%, 24.2%) | RR: 0.62 (0.38, 1.00)  RD: -0.095 (-0.172, -0.019) | aRR^1^: 0.63 (0.39, 1.01)  aRD^1^: -0.094 (-0.172, -0.164) |  |
|  | Inflammatory Conditions | 56/223  25.1% (19.6%, 31.3%) | RR: 1.00 (0.78, 1.29)  RD: 0.001 (-0.062, 0.064) | aRR^1^: 0.95 (0.74, 1.12)  aRD^1^: 0.012 (-0.074, 0.049) |  |
|  | Locking/Instability | 8/39  20.5% (9.3%, 36.5%) | RR: 0.82 (0.44, 1.54)  RD: -0.045 (-0.174, 0.085) | aRR^1^: 0.84 (0.45, 1.55)  aRD^1^: -0.042 (-0.173, 0.089) |  |
|  | Neurological Deficit | 7/24  29.2% (12.6%, 51.1%) | RR: 1.17 (0.62, 2.20)  RD: 0.042 (-0.142, 0.226) | aRR^1^: 1.13 (0.60, 2.14)  aRD^1^: 0.034 (-0.147, 0.214) |  |
|  | Operative Planning | 16/75  21.3% (12.7%, 32.3%) | RR: 0.85 (0.54, 1.34)  RD: -0.037 (-0.133, 0.060) | aRR^1^: 0.87 (0.56, 1.35)  aRD^1^: -0.034 (-0.131, 0.064) |  |
|  | Tumour | 34/169  20.1% (14.4%, 27.0%) | RR: 0.81 (0.59, 1.11)  RD: -0.049 (-0.115, 0.017) | aRR^1^: 0.74 (0.54, 1.03)  aRD^1^: -0.065 (-0.128, -0.001) |  |
| Reporting Grade | |  |  |  |  |
|  | Consultant only | 368/1838  20.0%  (18.2%, 21.9%) | Reference | Reference |  |
|  | Co-reporting consultant and trainee | 85/270  31.5% (26.0%, 37.4%) | RR: 1.57 (1.29, 1.92)  RD: 0.115 (0.056, 0.173) | aRR^2^: 1.52 (1.25, 1.86)  aRD^2^: 0.106 (0.048, 0.163) |  |
|  | Reported by trainee, reviewed by consultant | 74/217  34.1% (27.8%, 40.8%) | RR: 1.70 (1.39, 2.09)  RD: 0.141 (0.075, 0.206) | aRR^2^: 1.62 (1.32, 1.99)  aRD^2^: 0.126 (0.062, 0.189) |  |
| Field Strength | |  |  |  |  |
|  | 1.5T | 393/1838  21.4% (19.5%, 23.3%) | Reference | Reference |  |
|  | 3T | 134/444  30.1% (25.9%, 34.7%) | RR: 1.41 (1.20, 1.67)  RD: 0.088 (0.041, 0.135) | aRR^3^: 1.28 (1.08, 1.52)  aRD^3^: 0.062 (0.016, 0.107) |  |
|  | Missing | 0/43 | - | - |  |
| Contrast Enhanced Scan | |  |  |  |  |
|  | No^5^ | 467/1992  23.4% (21.6%, 25.4%) | Reference | Reference |  |
|  | Yes | 60/333  18.0% (14.0%, 22.6%) | RR: 0.77 (0.60, 0.98)  RD: -0.054 (-0.100, -0.009) | aRR^4^: 0.84 (0.66, 1.07)  aRD^4^: -0.037 (-0.086, 0.012) |  |
| RR: Risk Ratio RD: Risk Difference aRR: Adjusted Risk Ratio aRD: Adjusted Risk Difference ^1^Adjusted for age and sex ^2^Adjusted for age, indication, anatomical location, and field strength ^3^Adjusted for age, indication, anatomical location, and reporting grade ^4^Adjusted for indication and anatomical location  ^5^Where contrast wasn’t reported, no has been assumed | | | | |  |

Supplementary Table 1: Risk of at least one incidental finding (scan started population) by strata
