# Supplementary material for: Incidental findings associated with MRI of the hand and wrist
Source: Br J Radiol. 2025 Aug 12;98(1175):1997–2004. doi: 10.1093/bjr/tqaf194 (PMC12659746; doi:10.1093/bjr/tqaf194)
Supplement: tqaf194_Supplementary_Data [file tqaf194_supplementary_data.zip › Supplementary table 2 only.docx]

|  |  | Incidentalomas /Total Scans  % (95% CI) | Unadjusted Estimate (95% CI) | Adjusted Estimate (95% CI) |  |
| --- | --- | --- | --- | --- | --- |
| Overall | | 73/2325  3.1% (2.5%, 3.9%) |  |  |  |
| Age | |  |  |  |  |
|  | Per additional 10 years |  | RR: 1.09 (0.96, 1.24)  RD: 0.003 (-0.001, 0.007) |  |  |
| Sex | |  |  |  |  |
|  | Female | 40/1208  3.3% (2.4%, 4.5%) | Reference |  |  |
|  | Male | 33/1117  3.0% (2.0%, 4.1%) | RR: 0.89 (0.57, 1.40)  RD: -0.004 (-0.018, 0.011) |  |  |
| Anatomical Location | |  |  |  |  |
|  | Hand only | 13/340  3.8% (2.1%, 6.4%) | Reference | Reference |  |
|  | Wrist only | 60/1960  3.1% (2.3%, 3.9%) | RR: 0.80 (0.45, 1.44)  RD: -0.008 (-0.029, 0.014) | aRR^1^: 0.83 (0.46, 1.49)  aRD^1^: -0.006 (-0.028, 0.015) |  |
|  | Hand and Wrist | 0/25  0.0% (0.0%, 13.7%) | RR: Not Computable  RD: -0.038 (-0.059, -0.018) | aRR^1^: Not Computable  aRD^1^: -0.037 (-0.057, -0.017) |  |
| Indication | |  |  |  |  |
|  | Trauma | 38/976  3.9% (2.8%, 5.3%) | Reference | Reference |  |
|  | Further Imaging | 14/722  1.9% (1.1%, 3.2%) | RR: 0.50 (0.27, 0.91)  RD: -0.020 (-0.035, -0.004) | aRR^1^: 0.50 (0.28, 0.92)  aRD^1^: -0.019 (-0.035, -0.004) |  |
|  | Haematological/Vascular Pathology | 4/97  4.1% (1.1%, 10.2%) | RR: 1.06 (0.39, 2.90)  RD: 0.002 (-0.039, 0.044) | aRR^1^: 1.07 (0.39, 2.93)  aRD^1^: 0.003 (-0.039, 0.045) |  |
|  | Inflammatory Conditions | 6/223  2.7% (1.0%, 5.8%) | RR: 0.69 (0.30, 1.61)  RD: -0.012 (-0.036, 0.012) | aRR^1^: 0.66 (0.28, 1.54)  aRD^1^: -0.014 (-0.037, 0.010) |  |
|  | Locking/Instability | 2/39  5.1% (0.6%, 17.3%) | RR: 1.32 (0.33, 5.26)  RD: 0.012 (-0.058, 0.083) | aRR^1^: 1.34 (0.34, 5.33)  aRD^1^: 0.013 (-0.058, 0.085) |  |
|  | Neurological Deficit | 0/24  0.0% (0.0%, 14.2%) | RR: Not Computable  RD: -0.039 (-0.051, -0.027) | aRR^1^: Not Computable  aRD^1^: -0.039 (-0.051, -0.027) |  |
|  | Operative Planning | 3/75  4.0% (0.8%, 11.2%) | RR: 1.03 (0.36, 3.25)  RD: 0.001 (-0.045, 0.047) | aRR^1^: 1.05 (0.33, 3.30)  aRD^1^: 0.002 (-0.045, 0.049) |  |
|  | Tumour | 6/169  3.6% (1.3%, 7.6%) | RR: 0.91 (0.39, 2.12)  RD: -0.003 (-0.034, 0.027) | aRR^1^: 0.85 (0.36, 2.00)  aRD^1^: -0.006 (-0.035, 0.023) |  |
| Reporting Grade | |  |  |  |  |
|  | Consultant only | 48/1838  2.6% (1.9%, 3.4%) | Reference | Reference |  |
|  | Co-reporting consultant and trainee | 12/270  4.4% (2.3%, 7.6%) | RR: 1.70 (0.92, 3.16)  RD: 0.018 (-0.007, 0.044) | aRR^2^: 1.73 (0.92, 3.23)  aRD^2^: 0.019 (-0.007, 0.045) |  |
|  | Reported by trainee, reviewed by consultant | 13/217  6.0% (3.2%, 10.0%) | RR: 2.29 (1.26, 4.17)  RD: 0.034 (0.001, 0.066) | aRR^2^: 2.24 (1.22, 4.10)  aRD^2^: 0.032 (0.000, 0.065) |  |
| Field Strength | |  |  |  |  |
|  | 1.5T | 55/1838  3.0% (2.3%, 3.9%) | Reference | Reference |  |
|  | 3T | 18/444  4.1% (2.4%, 6.3%) | RR: 1.35 (0.80, 2.28)  RD: 0.011 (-0.009, 0.031) | aRR^3^: 1.28 (0.75, 2.18)  aRD^3^: 0.009 (-0.011, 0.028) |  |
|  | Missing | 0/43 | - | - |  |
| Contrast Enhanced Scan | |  |  |  |  |
|  | No^5^ | 69/1992  3.5% (2.7%, 4.4%) | Reference | Reference |  |
|  | Yes | 4/333  1.2% (0.3%, 3.0%) | RR: 0.35 (0.13, 0.94)  RD: -0.023 (-0.037, -0.008) | aRR^4^: 0.34 (0.12, 0.93)  aRD^4^: -0.023 (-0.037, -0.009) |  |
| RR: Risk Ratio RD: Risk Difference aRR: Adjusted Risk Ratio aRD: Adjusted Risk Difference ^1^Adjusted for age and sex ^2^Adjusted for age, indication, anatomical location, and field strength ^3^Adjusted for age, indication, anatomical location, and reporting grade ^4^Adjusted for indication and anatomical location  ^5^Where contrast wasn’t reported, no has been assumed | | | | |  |

Supplementary Table 2: Risk of incidentalomas (scan started population) by strata
