# Supplementary material for: Incidental findings associated with MRI of the hand and wrist
Source: Br J Radiol. 2025 Aug 12;98(1175):1997–2004. doi: 10.1093/bjr/tqaf194 (PMC12659746; doi:10.1093/bjr/tqaf194)
Supplement: tqaf194_Supplementary_Data [file tqaf194_supplementary_data.zip › Supplementary Figure 1 only.pdf]

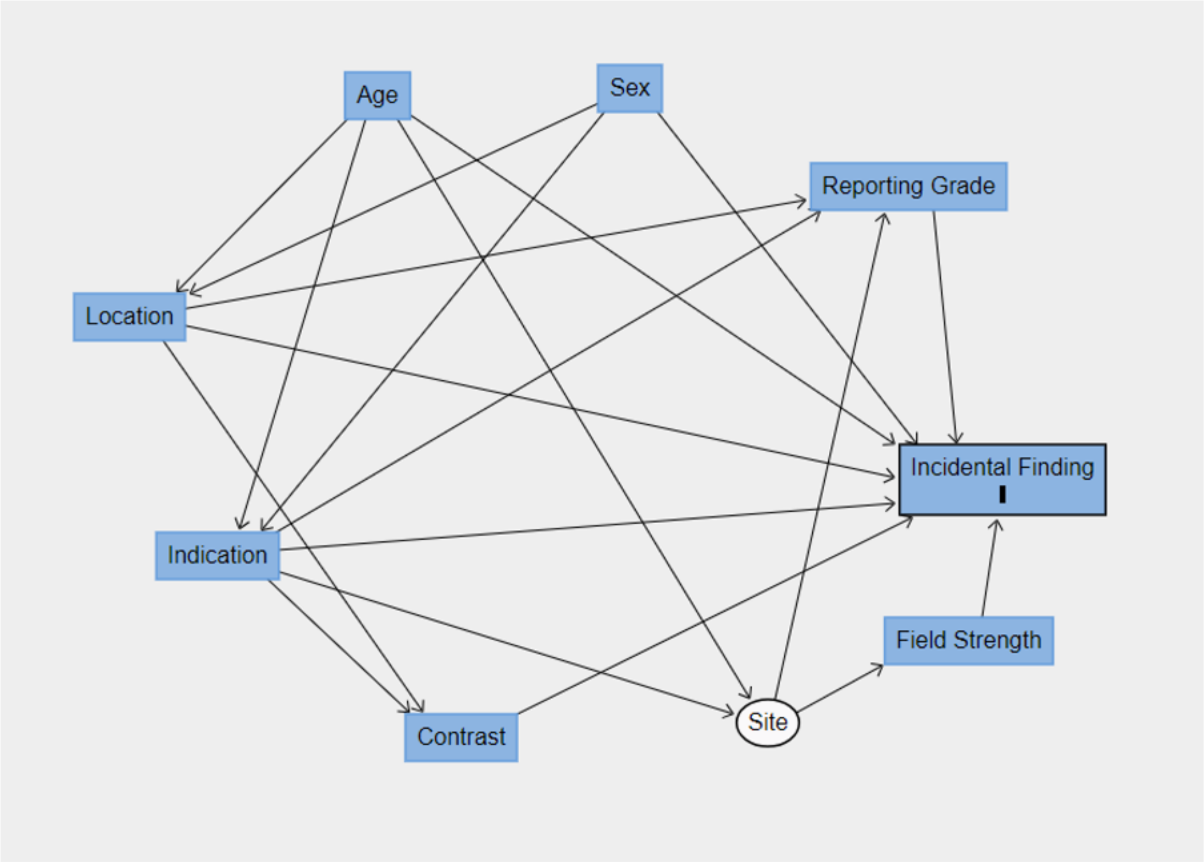

Supplementary Figure 1: This DAG was utilised to select the variables necessary to adjust the models.
